# Spatial clustering of hosts can favor specialist parasites

**DOI:** 10.1002/ece3.70273

**Published:** 2024-11-17

**Authors:** Jeremy Draghi, Evan Zook

**Affiliations:** ^1^ Department of Biological Sciences Virginia Tech Blacksburg Virginia USA

**Keywords:** host–parasite, modeling, niche evolution, optimal foraging theory, phage

## Abstract

Generalist parasites seem to enjoy the clear ecological advantage of a greater chance to find a host, and genetic trade‐offs are therefore often invoked to explain why specialists can coexist with or outcompete generalists. Here we develop an alternative perspective based on optimal foraging theory to explain why spatial clustering can favor specialists even without genetic trade‐offs. Using analytical and simulation models inspired by bacteriophage, we examine the optimal use of two hosts, one yielding greater reproductive success for the parasite than the other. We find that a phage may optimally ignore the worse host when the two hosts are clustered together in dense, ephemeral patches. We model conditions that enhance or reduce this selective benefit to a specialist parasite and show that it is eliminated entirely when the hosts occur only in separate patches. These results show that specialists can be favored even when trade‐offs are weak or absent and emphasize the importance of spatiotemporal heterogeneity in models of optimal niche breadth.

## Graphical abstract

Specialist parasites are common despite some clear advantages to a broader host range. Here we develop an optimal foraging model to explain why spatial clustering can favor specialists even without genetic trade‐offs. We find that a parasite may optimally ignore the worse host when the two hosts are jointly clustered in dense, ephemeral patches, even when trade‐offs are weak or absent.

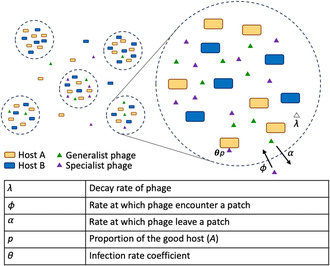

## 1 INTRODUCTION

A parasite's host range—the species, and host genotypes within each species, that a parasite can productively infect—is subject to strong and often conflicting selection pressures (Leggett et al., 2013; Longdon et al., 2014; Visher & Boots, 2020). Parasites that are poorly adapted to a host may suffer the fitness costs of nonoptimal virulence, vulnerability to host defenses, or poor competitive ability against other parasites. Adapting to a host in order to minimize these costs may favor specialization on one or a few host types. However, parasite fitness may also be limited by infection opportunities, which might favor a broader host range. Adaptive evolution in parasites resolves these tensions in varied ways, resulting in a diversity of coexisting generalist and specialist parasites in many ecosystems. Theory has contributed to our understanding of host‐range evolution, highlighting, for example, the role of dispersal and mutation rate in pathogen spillover (Gandon et al., 2013). However, we are still unable to make a comprehensive prediction of how the ecology and life history characteristics of a given parasite shape its evolutionary trajectory toward a broad or narrow host range.

Theory has focused mainly on how genetic trade‐offs—hypothesized limits dictating that high performance on one host must be accompanied by submaximal performance on another—shape optimal host range. Parasites often have small genomes and highly specialized phenotypic adaptations to particular hosts, supporting the intuitive notion that their host ranges are constrained by trade‐offs. However, trade‐offs have proven inadequate as the sole explanation for variation, and specialization, in parasite host ranges (Gompert et al., 2015). Evolution experiments comparing viruses adapting to a single host against populations adapting to multiple hosts find mixed and generally inconsistent evidence for strong trade‐offs (Bono et al., 2017; Remold, 2012; Turner & Elena, 2000). This pattern is echoed in a broader literature examining microbial trade‐offs across environments (Leiby & Marx, 2014; McGee et al., 2014; Rodríguez‐Verdugo et al., 2014; Sackman & Rokyta, 2019; Satterwhite & Cooper, 2015). Often, tradeoffs are present on average but appear highly variable in individual clones or replicate populations (e.g., Sant et al., 2021) or vary with factors like population size (Chavhan et al., 2021). These results suggest that the constraining effects of trade‐offs might have been avoided if evolution had taken other paths. Trade‐offs may also be present but masked by other factors such as variation in additional aspects of performance (Pease & Bull, 1988; Pinheiro et al., 2016), increasing the difficulty of predicting constraints on host range from trade‐offs. These limitations of the trade‐off model have driven interest in alternative explanations for why, and under what circumstances, parasites might benefit from specialized host ranges.

Optimal foraging theory provides a complementary approach to understanding how natural selection might act on niche breadth. As the name implies, optimal foraging theory is based on optimizing a measure of fitness, or a proxy for fitness such as the rate of caloric intake, over a range of possible foraging strategies (Charnov, 1976; Pyke, 1984). While typically applied to animal behavior, the theory is equally applicable to parasites, even those lacking any semblance of plastic behavior. Optimal foraging approaches are rooted in the same principle of fitness maximization as explanations based in trade‐offs, yet traditionally focus more on how the details of complex ecologies may drive differences in optimal niche breadth. As such, these approaches may yield complementary insights to analyses of trade‐off relationships.

Optimal foraging theory has been applied to diverse parasites such botflies (Manzoli et al., 2021), parasitic plants (Koch et al., 2004) and parasitoid wasps (Hubbard & Cook, 1978), but phage—viral pathogens of bacteria—have particularly inspired application and testing of these ideas (Abedon et al., 2003; Bull & Wang, 2010; Eshelman et al., 2010; Guyader & Burch, 2008; Heineman et al., 2008; Heineman & Bull, 2007; Hesse et al., 2015; Kannoly et al., 2022; Roychoudhury et al., 2014; Wang, 2006). Bull (2006) established a simple rule for optimal host range in lytic phage populations based on the burst size—the expected number of viable progeny—that they achieve on various hosts. Namely, for a viral population of constant size it is optimal to infect any host whose effective burst size (i.e., the burst size devalued by the probability of host death before lysis) exceeds one. Disregarding trade‐offs, then, it seems that the optimal host range would be expected to include all productive hosts.

To see this result intuitively, first consider a specialist phage population at ecological equilibrium with host A; the average reproductive success of each phage particle must therefore be one. Now, imagine a phage particle encountering an individual of host B. It is optimal to infect host B if the expected fitness payoff exceeds the opportunity cost of the choice, which is the product of the expected reproductive success on host A and the probability of finding host A before decay if host B is ignored. Since we have established that this product is one under the assumption of demographic equilibrium, that too is the threshold for the burst size of a minimally useful alternative host. While this low threshold seems to broadly favor generalists, host defenses, like CRISPR, that have the capacity to inhibit infection can substantially reduce the average burst size, potentially bringing it near one (Sieber & Gudelj, 2014). Still, this analysis seems to deepen the puzzle of why parasites are sometimes specialized.

The above prediction of an optimally broad host range assumes demographic equilibrium, and phage have been predicted to be optimally more specialized when their populations are growing (Bull, 2006; Guyader & Burch, 2008). These results suggest that parasite specialization might be predicted by optimal foraging theory in circumstances in which the opportunity cost of infecting a host with a marginal payoff is elevated above one. Here, we analyze a model in which two hosts, differing in the average fecundity of infections, are structured in dense but ephemeral clusters. Such high‐density clusters, representing patches of resource from the parasite's perspective, can arise when hosts temporarily aggregate around food resources. For example, marine bacteria may seek out and colonize food particles in the water column (Stocker et al., 2008), later detaching after consuming most of the available resources (Yawata et al., 2020).

Using mathematical and simulation models, we show that the joint aggregation of two hosts into patches can greatly increase the opportunity cost of infecting the lower‐quality host, consequently selecting for specialization even when the expected productivity of infecting the worse host is much higher than one. This high opportunity cost arises because parasites that encounter an individual of the worse host would, if they rejected it, have a high chance of infecting the better host by virtue of the spatial proximity of the two host types. We first illustrate these ideas with a simple mathematical treatment of within‐patch fitness, validated over violations of some assumptions with numerical methods. We then explore a complex, epidemiological simulation model of phage/host dynamics to show that opportunity cost in mixed‐host patches can predict conditions that favor specialist phage. These results show that spatial structure of hosts can strongly favor specialists, even in the absence of any genetic trade‐offs on performance across hosts.

## 2 MODEL & RESULTS

### 2.1 Modeling parasite fitness in a heterogeneous host patch

To obtain a preliminary understanding of the selective forces on parasite host ranges within a metapopulation of host patches, we first consider how host‐range affects reproductive success for a parasite within a patch containing two types of hosts. Although the principles we model are likely to be applicable to a range of organisms, for concreteness we focus on the biology of bacteriophages (“phage”)—viral parasites of bacterial hosts. Our approach is to consider a generalist phage, able to infect either of two hosts, A or B, in comparison to a specialist phage which only infects host A. We assume no trade‐offs in performance on a host, such that the generalist and specialist are equally capable of infecting and using host A. We also assume that these infection strategies, along with all other traits of the phage and the infections they create, are fixed, rather than plastic. We define phage fecundity on each host as the average number of infectious virions produced from a successful infection, labeled as *F*_A_ and *F*_B_ for the two hosts A and B. Throughout, we define A to be the better host such that *F*_A_ *> F*_B_.

Our model focuses on spatial clustering of hosts, such as might occur if hosts aggregate and grow around resource particles, then disperse after exhaustion of local resources. We consider an aggregation as a “patch” of resources, mirroring the typical terminology of optimal foraging approaches. Spatial structure is simplified to a binary state: a phage is either inside a patch or outside any of the patches, with no further details of its location specified. Furthermore, we assume that the period between infection and lytic release of progeny—the burst time (*L*)—is longer than the remaining lifetime of the aggregation. Because of this latter assumption, the progeny of any infection generated by a phage inside a patch will emerge into the low‐host‐density world between patches, rather than the prime reproductive opportunity of a host aggregation; the effects of relaxing this assumption are evaluated later.

We first consider the fate of a generalist phage particle located in a patch: it might infect either type of host, decay, or exit the patch via diffusion. Let *α* represent the rate at which a phage particle exits the patch, *λ* represent the decay rate, and *θ* represent the rate of potentially infectious contacts between the phage and a host. A fraction *p* of those contacts occurs with host A and 1 − *p* with host B. We assume that these proportions are invariant and relax this assumption in numerical results below.

With these assumptions, we compute the expected reproductive success of a generalist phage within a patch as:

(1) $$W_{G}=\frac{\mathrm{\theta p}F_{A}+\theta \left(1-p\right)F_{B}+\alpha }{\alpha +\theta +\lambda }$$

This equation is simply a weighted average of the output of phage from the patch. For example, *θ p*/(*α* + *θ* + *λ*) is the probability that the phage infects host A, in which case an average of *F*_A_ phage are produced. We equate phage produced by an infection with those exiting the patch via diffusion because we assume that infections burst only after a delay, and therefore do so when the cells are outside the patch. In doing so, we ignore any difference in fitness stemming from the timing with which a phage enters the space between patches.

The equation for the specialist differs from Equation 1 in two ways: the term (1 − *p*) *F*_B_ is absent from the numerator, decreasing fitness, and the denominator is decreased to *α* + *θp* + *λ*, increasing fitness.

(2) $$W_{S}=\frac{\mathrm{\theta p}F_{A}+\alpha }{\alpha +\mathrm{\theta p}+\lambda }$$

We next solve *W*_*G*_ = *W*_*S*_ in order to find the boundary between parameters favoring generalists and those favoring specialists, yielding $F_{B}\;\left(\alpha +\mathrm{\theta p}+\lambda \right)=\mathrm{\theta p}F_{A}+\alpha$. Further simplification can be made by substituting the net fecundities, *n*_A_ = *F*_A_ − 1 and *n*_*B*_ = *F*_B_ − 1. These net values account for the loss of the parental phage, which is destroyed during viral reproduction.

(3) $$\begin{array}{c} n_{B}+1=\frac{\mathrm{\theta p}}{\alpha +\mathrm{\theta p}+\lambda }n_{A}+\frac{\alpha +\mathrm{\theta p}}{\alpha +\mathrm{\theta p}+\lambda }\\ n_{B}=\frac{n_{A}\mathrm{\theta p}-\lambda }{\alpha +\mathrm{\theta p}+\lambda } \end{array}$$

If we were to set *α* = 0, we would remove migration and could therefore consider Equation 3 as modeling a single, well‐mixed population. If we further specify *n*_*A*_ *θp* = *λ*, thereby ensuring that the phage population is at demographic equilibrium, we can recover the prior results from Bull (2006): generalists and specialists have equal fitness when *n*_B_ = 0.

We can simplify Equation 3 by assuming that most phage will decay before finding a patch. Therefore, demographic equilibrium requires that, on average, a phage that does find a patch will reproduce far in excess of its death rate (*n*_*A*_ *θp* ≫ *λ*). Ignoring *λ* in the numerator yields a useful approximation:

(4) $$n_{B}\approx \frac{n_{A}\mathrm{\theta p}}{\alpha +\mathrm{\theta p}+\lambda }$$

Using Equation 4, we can write the ratio *n*_*A*_ and *n*_*B*_ that equalizes specialist and generalist fitnesses as:

(5) $$R^{*}=\frac{n_{B}}{n_{A}}\approx \frac{\mathrm{\theta p}}{\alpha +\mathrm{\theta p}+\lambda }$$

We interpret R* as an indicator of how profitable the worse host must be to make generalists equally to specialists. If R* is, for example, 0.5, then we predict that specialists should be favored unless the worse host offers at least 50% of the expected fecundity of the better host. Specialists are highly favored as R* approaches one, which occurs when the rate at which a specialist finds the better host (*θp*) is large compared to the chances of leaving the patch or decaying. In contrast, small values of R* result when the chance of finding the preferred host are small. We can interpret this result as indicating that choosiness—host specialization—is disfavored when phage are better off infecting the first host they encounter.

Equivalently, $\frac{n_{A}\mathrm{\theta p}}{\alpha +\mathrm{\theta p}+\lambda }$ can be viewed as the opportunity cost paid by a generalist phage that infects host B, rather than behaving like the specialist and rejecting host B. As used here, the opportunity cost measures the expected value of an alternative action, mutually exclusive with the action of infecting host B. Our Equation 4 therefore expresses that generalists and specialists have equal fitness when this opportunity cost is equal to the expected net reproductive success, *n*_*B*_, achieved by infecting host B.

In order to test these predictions, we performed individual‐based simulations phage reproduction in a single patch filled with both types of hosts (see Section 4.1). Host dynamics in these simulations matches several assumptions of the analytic model—hosts were present at fixed proportions that were not perturbed by infections, and we did not explicitly model the breakdown of the aggregation. Figure 2 shows that our analytic predictions match simulation results under these assumptions. A visual example of how our simulation finds R* can be seen in Figure S1: the intersection point of the two fitness sets marks the ratio predicted by Equation 5. In Figure 2a, we illustrate how increasing the rate of infectious contact with hosts or the frequency of the good host favors specialists. In 2B, we highlight how increasing the rate at which phage leave aggregations or the rate at which they decay favors generalists. These relationships can all be understood with one principle: increasing the chance that a phage would find host A after encountering, and rejecting, host B favors host‐A specialists.

We used this simple simulation framework to test the robustness of our results to two assumptions: that infection did not burst within a patch, and that both hosts were present in uniform proportions across patches. To understand the role of secondary infections, a new parameter, *ω*, was introduced, representing the probability that an infected host will burst inside the patch. Across various values of *ω*, our predictions do not greatly deviate from the simulation (Figure 3a). We also simulated variation in the frequencies of each host within a patch by setting an effective number of hosts, and drawing values of *p* for each simulated replicate from a binomial distribution with the standard deviation appropriate to that size. As shown in Figure 3b, our predictions remain accurate except that we overestimate R* at very small patch sizes. This overestimation is to be expected because variability in very small patches may result in patches with only one host, violating our core assumption that phage are confronted with mixed patches.

### 2.2 Simulating the generalist and specialist performance in ephemeral host patches

**FIGURE 1:**
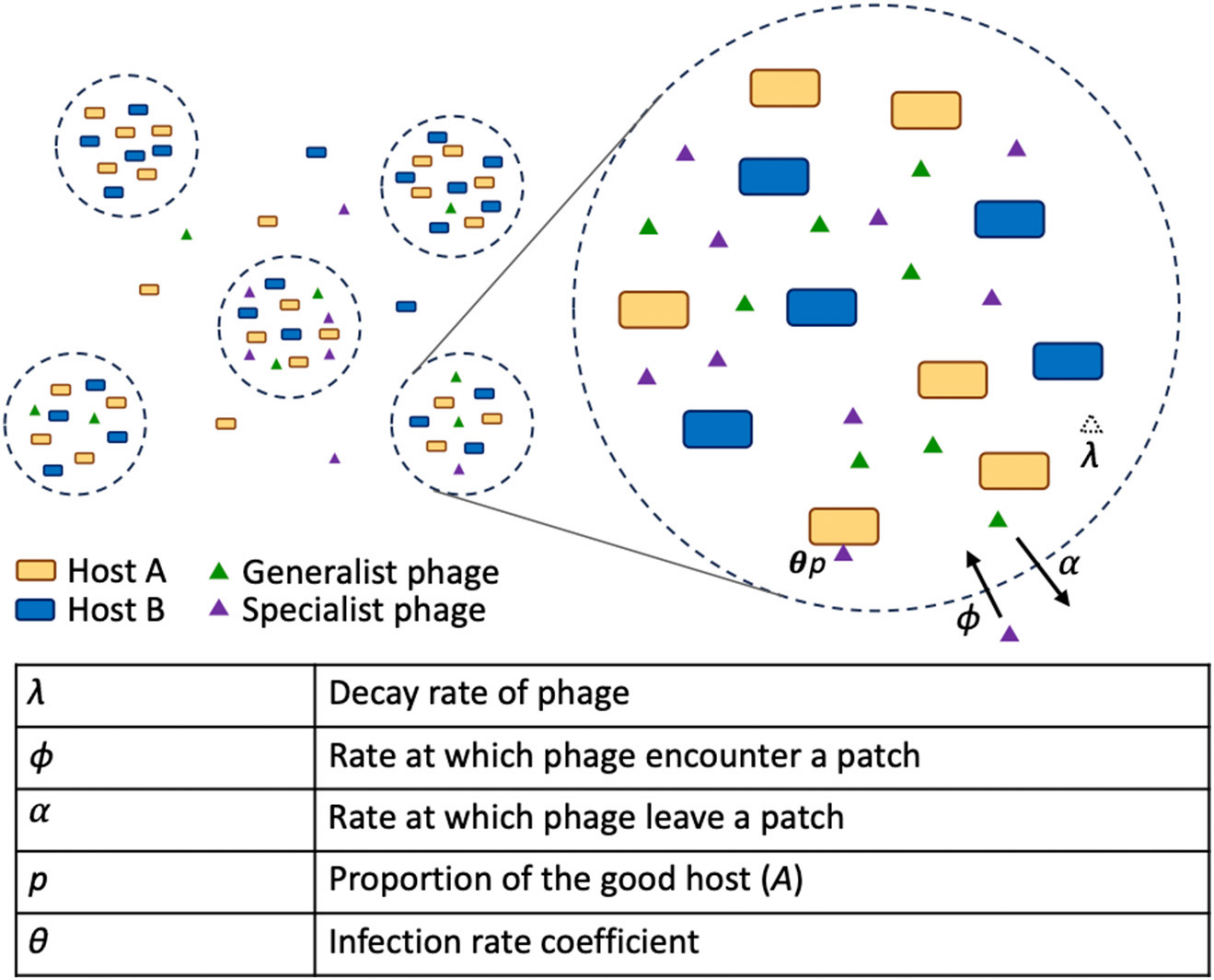
A simple diagram showing how our model represents a patch and its occupants. Hosts A and B (rectangles) and specialist and generalist phage (triangles) aggregate in patches and undergo decay, interact via infection, and communicate with the outside world via migration.

The modeling above suggests that specific ecological circumstances could favor specialists of host A, even if host B is only slightly less valuable. However, violations to several simplifying assumptions could undermine or otherwise complicate this prediction. First, within the dynamic metapopulation of patches pictured above in Figure 1, the demographics of generalist and specialist phage and their hosts would be linked through competition in complex ways. These dynamics would ultimately determine whether specialist or generalist parasites could outcompete the other within an epidemiological arena. Second, host aggregations themselves would be highly dynamic. Without infection, we expect cell populations on a food particle to grow approximately exponentially, via both immigration from planktonic cells and growth within the aggregation, then diminish as cells resources are exhausted and cells detach. In the presence of infection, these dynamics would only increase in complexity and heterogeneity. Also, phage might infect a cell inside an aggregation and burst before that aggregation disperses. The simulations above assess how these secondary infections would directly affect fitness, but disregard how such infections would feed back on the densities of hosts and the lifetime of the patch itself. To counter this shortcoming, we developed a complex simulation which keeps track of population dynamics within a pool of patches, each similar to that shown in Figure 1. These dynamics ultimately determine how often a generalist phage encounters a mixed patch and actually pays an opportunity cost for its broader host range.

To explore the effects of these ecological complexities, we designed a simulation model of individual patches. Each patch begins with set amounts of resources, with the distribution of those resources defining the treatment imposed in our simulated experiments. In the *together* treatment, each patch is furnished with equals amounts of two resources, *R*_A_ and *R*_B_. Each resource is exclusively consumed by either host A or host B. In the *apart* treatment, each patch contains only *R*_A_ or *R*_B_, with equal probability. Therefore, we use the composition of the resource patches to control the landscape of coinciding hosts that forms the selective environment for phage. New resources only enter the system when patches arise. Hosts arrive from an external pool in which the patches are suspended, settling in patches which contain some quantity of their relevant resource; once settled, they consume resources, reproduce, and leave the aggregation as resources deplete. Phage also arrive from the external pool and can infect cells, decay, or leave again via diffusion. Infected cells do not grow but may die, leave, or burst, releasing phage progeny. Phage and cells in the pool may move into patches or decay, but do not otherwise interact; as assumed above, no infections arise in the pool. Simulations use a modified version of the Gillespie algorithm (Gillespie, 1977); further details are described in *Computational Methods*, and Table 1 defines and lists all parameters of this expanded model.

**TABLE 1:** Descriptions and default values for simulation model parameters in part II. The third column indicates whether this parameter was held constant or varied across a range during evaluation of parameter sets.

Parameter	Description	Default value
*Y*	Resource abundance, in units of host cell yield	200
*v*	Rate coefficient for host cells arriving at a given aggregation (h^−1^)	Varied
*r*_*A*_	Maximum host A division rate (h^−1^)	Varied
*r*_*B*_	Maximum host B division rate (h^−1^)	Varied
*K*	Half‐saturation constant for host growth	40
*d*	Rate coefficient for host cell detachment (h^−1^)	0.00167
*k*	Half‐saturation constant for host detachment	10
*σ*	Per capita resource decay rate (h^−1^)	8.33 × 10^−6^
*ϕ*	Rate coefficient for phage arriving at a given aggregation (h^−1^)	Varied
*α*	Per capita rate at which phage leave an aggregation (h^−1^)	Varied
*λ*_*p*_	Per capita decay rate of phage (h^−1^)	Varied
*λ*_*h*_	Per capita decay rate of hosts (h^−1^)	Varied
*θ*	Infection rate coefficient (h^−1^)	Varied
*χ*	Period between addition of new patches	Varied
*β*	Burst rate of infected cells (h^−1^)	8.33 × 10^−6^
*F*_A_	Mean fecundity of infections of host A	50
*F*_B_	Mean fecundity of infections of host B	Varied

To evaluate model behavior, we first find parameter sets that support stable, reasonably large populations of the generalist phage and both hosts. We varied nine parameters, each over orders of magnitude, to search for suitable parameter combinations representing a wide range of parameter space consistent with stable coexistence. Once a parameter set is classified as suitable (see Section 4 for details), we then conducted lengthy simulations in which both specialist and generalist phage are continuously introduced into the system via migration. Across these replicate simulations, we varied *F*_B_, the mean burst size of infections of host B by the generalist. Our goal was to find the value of *F*_B_ at which generalists and specialists have equal frequencies, indicating parity of fitness. We determined these breakeven values by fitting a generalized logistic model; details of the fitting process are discussed in the *Computational Methods*. We then translated this result into the R* ratio described above, which indicates how profitable, relative to the better host, the worse host must be in order for a generalist to be as fit as a specialist.

Figure 4 shows that our main result—specialists can be favored when good and bad hosts grow together—is validated in this more ecological complex model. The R* values for the *apart* treatment are closely centered around zero. This corresponds with the prediction derived from Bull (2006): in a steady‐state population, a host with a net productivity above zero should be included in the optimal host range. The results for the *together* treatment show much different outcomes; though there is considerable variability, for many parameter sets the specialist is superior unless the productivity of the worse host is a sizable fraction of the productivity of the better host.

In interpreting Figure 4, note that the *together* treatment pairs the two resources, which encourages but does not guarantee that the hosts will coincide. The variation evident in Figure 4 may therefore arise because patches are not effectively mixed under certain parameter combinations, thereby not actually presenting the generalist phage with a dilemma. We sought to see if this variation could be predicted by a measure of the opportunity cost, using modified simulations to ask whether a specialist phage that encountered an individual of host B would, after rejecting this host, survive to encounter host A. We describe this measure as *P*(A|B)—the conditional probability that host A is encountered after first encountering (but not infecting) host B. This measure integrates information about the spatial and temporal correlations between hosts as well as phage parameters like dispersal rates and lifespan. As shown in Figure 5, this conditional probability effectively predicts how favorable a parameter set will be in the *together* treatment for specialists. A linear regression yields an *R* ^2^ of .74; given that we measure opportunity costs in the absence of generalists, and therefore without consideration for their effects on the ecological equilibrium, the success of this prediction is notable.

## 3 DISCUSSION

**FIGURE 2:**
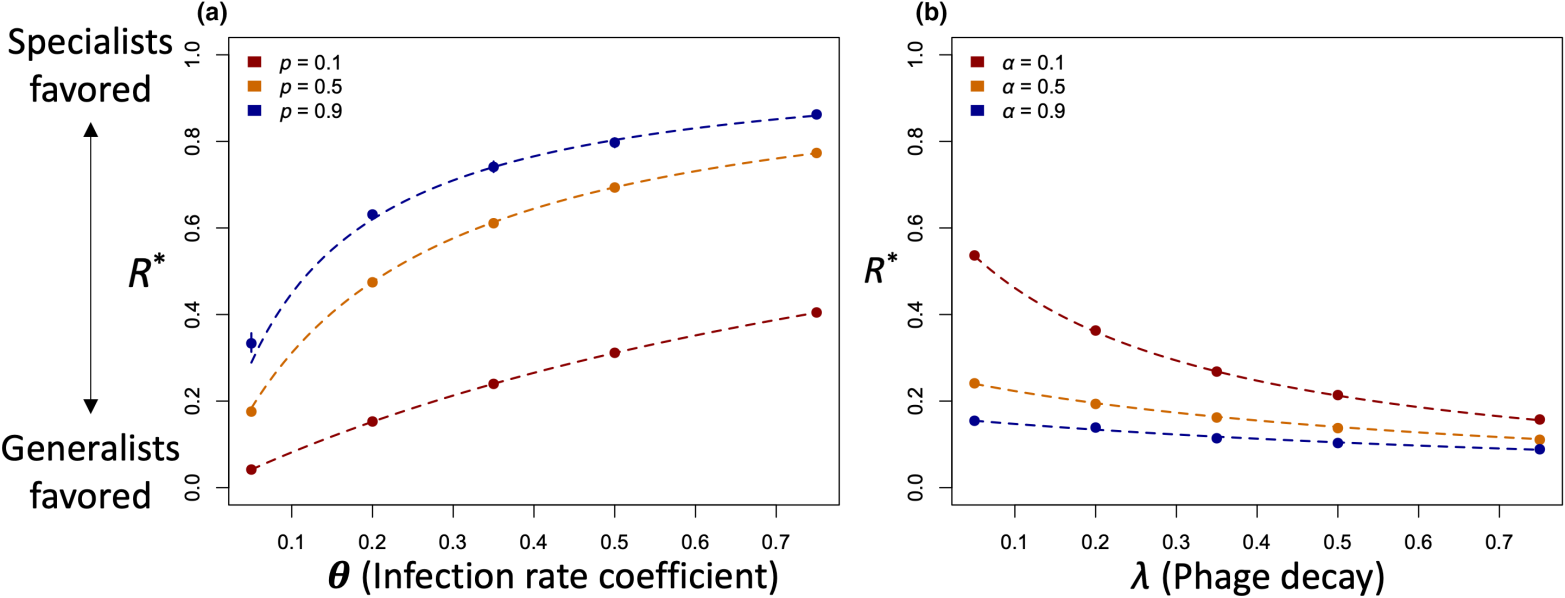
Single‐patch simulations match expectations from Equation 5. Panel (a) shows R* versus *θ* versus three different values of *p* with *α* at 0.1, *λ* at 0.01, and *ω* at 0. The points are simulated data, and the lines are the result of the mathematical prediction from Equation 5. Panel (b) shows R* versus *λ* with the same *ω*, a *p* of 0.5, and a *θ* of 0.35. Panel (a) displays parameters where the specialist is favored, and panel (b) shows the opposite. Each data point was simulated with 10^6^ trials. Confidence intervals, generated by bootstrapping, are visible where they exceed the size of the plotting symbols.

**FIGURE 3:**
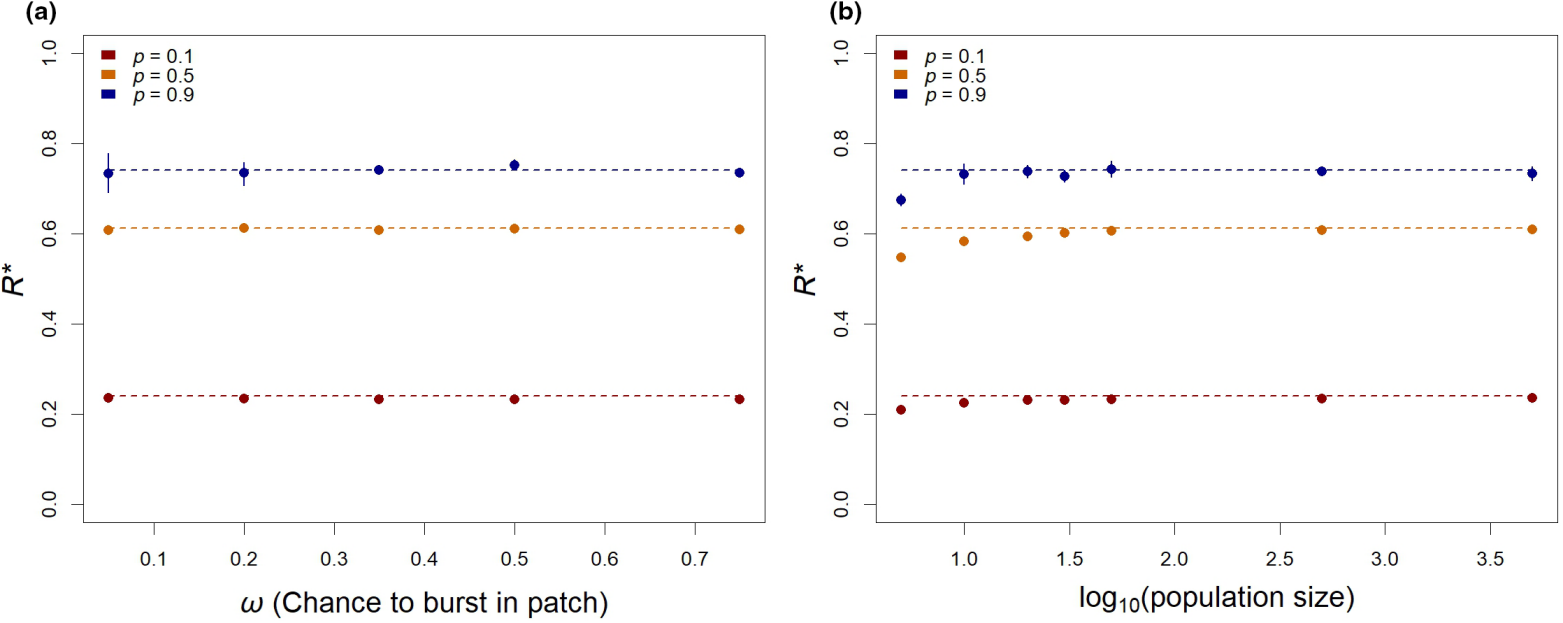
Predictions are robust to violations of two key assumptions. When infected cells have a chance to burst within the patch (*ω* > 0), values of R* remain as predicted (a; points are simulations, lines show Equation 5). Similarly, when patches were simulated with binomial variation in the frequency of each host, predictions remain consistent except for small effective sizes of patches (b). Each data point was simulated with 50^6^ trials.

**FIGURE 4:**
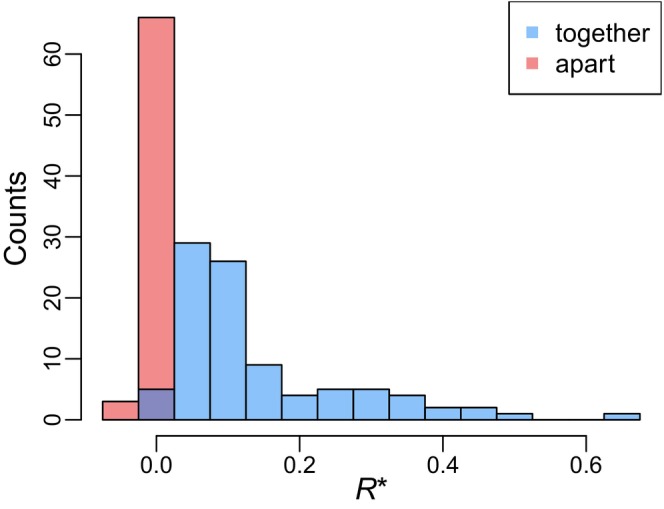
Relative advantage to specialists (quantified as R*) can be much higher when hosts occur together compared to when they occur separately in dynamic simulations of host patches. Histograms show inferred values of R*, defined as the relative net value of host B, compared to host A, at which generalists and specialists have equal fitness. A total of 93 parameter sets are shown in the *together* treatment; a subset of 69 found to be suitable in the *apart* treatment are plotted above. See Section 4 for details.

**FIGURE 5:**
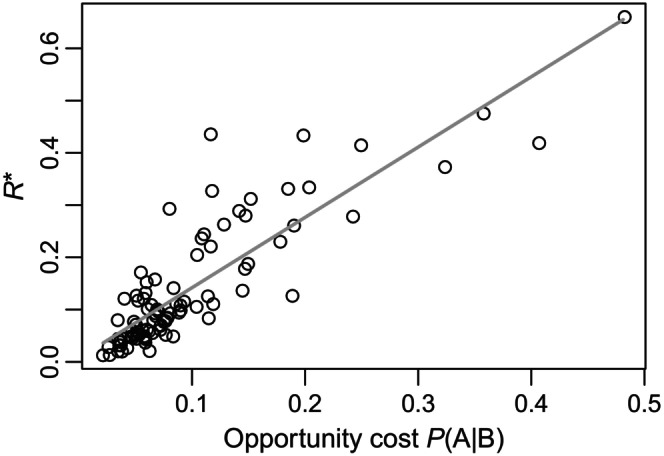
Relative advantage to specialists (quantified as R*) is well predicted by a measure of opportunity cost. Ninety‐three parameter sets from the *together* treatment are plotted. Opportunity cost is measured as the chance that a specialist phage that encounters a host‐B individual would go on to find a host‐A individual before decaying; see Section 4 for details.

In its most basic form, optimal foraging theory is concerned with choices among resources. Hosts may vary in the reproductive success they afford parasites, but our results show that the optimal host range is sensitive to other features such as the hosts’ distributions in space. Equation 5 predicts that specialist phage can be evolutionarily favored over generalist phage without trade‐offs, as illustrated in Figure 2. We show that this result holds across a range of deviations from our assumptions in Figure 3, and in a much more complex, dynamic model in Figure 4. In Figure 5, we show that specialists are favored when generalists face a genuine opportunity cost, quantified as the probability that a phage encountering host B would, if it rejected that inferior host, survive to encounter host A.

Together, these results support a model in which clustering of two hosts together in ephemeral patches rewards parasites that decline to infect the worse host. This selective effect becomes intuitive if modeled as if it were a decision made by an optimal forager. Specifically, it is optimal to reject an inferior resource if that rejection leads to a high chance that the forager would find a substantially better resource. In our models, that high chance of finding a good host after rejecting the bad arises by spatial clustering. This spatial proximity effectively makes contact with a poor host serve as a reliable signal indicating the nearby presence of good hosts. While phage and many other parasites may lack the capacity to process and respond to explicit signals, evolutionary competition between alternative strategies can still clarify the value of such information.

While our results may apply to many types of parasites, we have focused on bacteriophage due to the history of theoretical and experimental attention to optimal life histories in phage. As discussed in the *Introduction*, readily quantifiable phage traits such as lysis time and host range have provided opportunities for quantitative comparison with optimal foraging theory. Phage host ranges are readily mutable; one or a few mutations may cause phage to switch hosts (Duffy et al., 2007), infect a broader host range (Myers et al., 2023), or use a new host receptor (Meyer et al., 2012). This mutability has enabled direct probing, via experimental evolution, of the selective forces shaping competition between generalist and specialist phage (e.g., Bono et al., 2015). We hope that the results presented here will further stimulate the experimental test of theories regarding host‐range evolution.

Phage host ranges are also relevant to their roles in nature, via the control they exert on bacterial communities (Brown et al., 2022; Chevallereau et al., 2022), as well as in medicine. Increasing interest in using phage as therapeutics, to complement and potentially replace antibiotics, has driven the search for more effective ways to manipulate phage host ranges. A broad host range has both pros and cons for phage therapy (de Jonge et al., 2019): phage with a narrow host range may avoid some of the disruptive effects on microbiomes caused by broad‐spectrum antibiotics; on the other hand, broader host ranges allow doctors to prescribe effective treatments without the need for slow, expensive diagnostics. Theory that clarifies what ecological features most effectively select for broader or narrower host ranges can therefore contribute to the design of protocols to fine‐tune therapeutic phage (Bull et al., 2022).

Modern approaches to understanding niche breadth often focus on mechanisms shaping broad features of ecological networks (Pinheiro et al., 2016), in particular focusing on the broadly identified pattern of nestedness (Flores et al., 2011; Weitz et al., 2013). In host–parasite terms, this pattern is one in which the host range of a more specialized parasite is nested within the host range of generalist parasites. The challenge of understanding coexistence between generalists and specialists is then viewed through the lens of the origin and stability of nestedness (e.g., Leung & Weitz, 2016; Zografou et al., 2020). Further work could extend our results here to the context of a broader community, modeling how the effects of spatial structure in many host species might structure the infectivity network of their parasites.

Classical approaches to optimal foraging models often model patchy distributions of prey, and have noted that patches or clusters favor specialization, in comparison to more dispersed distributions, by virtue of decreasing mean search time for a preferred prey (Pulliam, 1974). Equally, the importance of spatial structure for processes like kin selection, antagonistic co‐evolution and the evolution of virulence has long been recognized in host–parasite ecology and evolution (Lion & Gandon, 2015). These mostly separate topics have sometimes been brought together in models connecting host range and virulence (Leggett et al., 2013) and in co‐evolution experiments, particularly those with *ϕ*2 and *Pseudomonas fluorescens* that combine antagonistic coevolution with host‐range evolution (Hall et al., 2011), dispersal (Lopez‐Pascua et al., 2010; Vogwill et al., 2008), or both (Hesse et al., 2015). Yet, we believe that the interactions between host distributions and selection on host range explored here have not previously been quantified. This novelty is notable because the evolution of host ranges, particularly in phage, is intently studied, as referenced in the *Introduction*. We note that the majority of phage‐host evolution experiments focus on exploring the complexity of genotype–fitness relationships against the relatively simple ecological background of well‐mixed environments. These decisions, which we speculate are motivated by the search for genetic trade‐offs, may limit the attention given to spatial structure. More recently, studies have found that spatial structure aids in long‐term coexistence of phage and bacterial hosts (Heilmann et al., 2012); it may be fruitful to extend such models to the questions of host‐range evolution considered here.

The models presented here are inspired by studies sketching out complex interactions among resource‐rich particles, aquatic bacteria, and bacteriophages; yet it should be acknowledged that these interactions are highly diverse and much remains to be characterized. Diverse bacteria colonize particles suspended in water with successional dynamics (Pascual‐Garcia et al., 2022), and there is a substantial literature studying chemotaxis, attachment and detachment from these particles from an optimal foraging perspective (Fernandez et al., 2019; Keegstra et al., 2022; Stocker, 2012; Taylor & Stocker, 2012; Yawata et al., 2020). One such study explicitly considered phage by modeling the trade‐off between increased growth and vulnerability to phage attack faced by aggregated bacteria (Ebrahimi et al., 2022). Our model assumes that particles nucleate bacterial aggregations that are ephemeral on the timescale of phage lysis and, in part II, that bacterial consumption is responsible for this fleeting association. While bacteria do not fully render large particles into biomass on short timescales, their enzymatic activity can rapidly degrade suspended particles (Smith et al., 1992). Suspended particles may, overall, act as unproductive sinks for phage (Weinbauer et al., 2009), but other studies point to potentially high rates of infection and mortality of particle‐associated bacteria via phage (Proctor & Fuhrman, 1991). Surface‐associated bacteria can also form biofilms that in some cases protect against phage (e.g., Vidakovic et al., 2018). The results here further motivate efforts to understand the diversity of microbial interactions in natural systems with a variety of distinct microenvironments.

A major assumption of our model is that parasites do not infect host cells outside of the context of patches. This assumption reflects our purpose: to quantify how spatial aggregation of hosts selects on host range. This assumption would be most realistic in scenarios in which parasites and hosts are at low densities or have limited/undirected dispersal outside of patches. We anticipate that allowing for parasite reproduction in other contexts, such as encounters between parasites and hosts outside of patches, would introduce a competing selective pressure in favor of generalists. Future work should certainly explore selective pressures in these more complex scenarios.

Our simulation model attempts to balance tractability with the inclusion of a fair degree of realism. Nonetheless, there is substantial room for future work to relax additional assumptions and incorporate more biology reflective of phage, and of parasites more broadly. For example, traits such as lysis time and mean fecundity can be plastic in phage, responding to host growth rate, host quality and resource availability (Abedon et al., 2001; Choua & Bonachela, 2019; Weitz & Dushoff, 2008)—incorporating this plasticity might change the relative payoffs of infecting poor hosts in our framework. Temperate phage, which integrate into host genomes or are replicated as extrachromosomal elements, would be expected to behave very differently from the strictly lytic phage we model here. Temperate phage can induce lytic infections in response to cue and manipulate host phenotypes as integrated prophages (Howard‐Varona et al., 2017), allowing for a more complex range of interactions between environments and phage traits than modeled here.

While the model here focuses entirely on optimal foraging behavior in parasites, the selection pressure exerted by parasites on their hosts also feeds back into optimal decisions by those hosts. In particular, we might expect that hosts leave patches earlier than predicted by resource availability, if this behavior reduced exposure to virulent parasites. The concept of a trade‐off between foraging success and exposure to threats has a long history in optimality theory (e.g., Abrams, 1993; Newman, 1991; Sih, 1980), including a specific model of particle‐associated bacteria and phage discussed above (Ebrahimi et al., 2022). Future modeling work could add this feedback into the basic framework developed here, allowing host behavior to co‐evolve with that of the phage parasites. Based on the results here, we might also predict that bacteria that already provide low expected payoffs to infecting phage, perhaps by virtue of defense mechanisms (Sieber & Gudelj, 2014), might achieve higher fitness by associating with preferred hosts; essentially, directing the evolution of their parasites in a favorable direction.

In summary, our modeling results have opened an investigation into a previously undescribed selective pressure connecting the spatial arrangement of hosts to the optimal host range of parasites. While previous work has established that parasites might optimally reject poor hosts, even without genetic trade‐offs, our results are the first to predict optimal rejection of hosts with substantial value to parasites (i.e., burst sizes much greater than one) outside of the context of an exponentially growing population. We would speculate that further theoretical attention to optimal parasites behavior in structured, steady‐state populations will continue to yield new insights into the complex selective pressures acting on host range.

## 4 COMPUTATIONAL METHODS

### 4.1 Methods for part 2.1

The focus of these individual‐based simulations is to highlight differences in fitness between generalists and specialists under varied parameters while inside a single aggregation. These simulations sampled from the simple probability distributions used to construct our equations: for example, a generalist phage left its aggregation with probability $\alpha /\left(\alpha +\theta +\lambda \right)$, decayed with probability $\lambda /\left(\alpha +\theta +\lambda \right)$, etc. To analyze how selection acted on host range, we found the burst size of host B, which yielded fitness equal to that of host A for each new set of parameters. By taking the ratio of the net burst size (burst size – 1) of host B and host A, we are able to compare parameter sets; see the discussion on R* above. Computationally, this was found by increasing the burst size of host B steadily from zero to just past the burst size of host A and subsequently simulating the fitnesses for both hosts. Thus, per each parameter set, the fitness of the specialist was a straight line and the fitness of the generalist had a positive slope. Upon analysis, this slope was found to be linear under normal parameters, allowing for simple approximation of the burst size of host B (see Figure S1 for an example).

Probabilistically, each phage can: leave the aggregation, giving it a fitness of one, die, giving it a fitness of zero, or infect a host, giving it a fitness dependent on the type of host it infects, and whether or not it bursts inside the aggregation. Should the phage infect a host and lyse inside the patch, the fitness of the infecting phage is determined by the reproductive success of its immediate progeny. To reduce random variance, we model the reproductive success of each of these secondary infections as equal to the expected burst size. If the host leaves the aggregation before bursting, then the phage will have a fitness equal to the host's burst size.

To find R*, we examine the simulated data sets to find nearby values of *F*_B_ for which *W*_*G*_ *> W*_*S*_ and *W*_*G*_ *< W*_*S*_. These points define intersecting lines, and we use linear regression to estimate the intersection point (Figure S1). Subtracting one from the *x*‐value of this intersection point, then dividing by the net burst size of host A yields our central estimate R*, as per Equation 5.

Confidence intervals were determined by re‐sampling with replacement to form bootstrapped data sets. To form a distribution of possible R* values, we perform regression and solve for the intersection point for each bootstrapped data set. This presents a technical problem, as bootstrapped data sets might produce lines that do not intersect, i.e., there is no breakeven point where the fitness of the generalist is equal to the specialist. When this problem occurs in reasonably small quantities (≤1%), we apply a swapping algorithm to the data. This algorithm works by changing which bootstrapped data sets are compared against each other and using random sampling to attempt to produce reordered sets of bootstrapped replicates that all meet the necessary criteria for linear regression.

### 4.2 Methods for part 2.2

#### 4.2.1 Simulation details

This simulation tracks the appearance, colonization, and decay of multiple resource patches suspended in a fluid medium. The dynamics of each patch are computed independently of other patches, interacting only through the immigration and emigration of phage and hosts with the surrounding medium. We do not track the locations of these patches, treating the contents of the surrounding medium as well‐mixed.

The overall simulation proceeds in cycles, each constituting a fixed interval—typically a 20‐min period of model time. Within each cycle, four types of events are processed in sequence: first internal patch dynamics, followed by decay of individuals in the medium, removal of exhausted patches and finally addition of new ones. First, the internal dynamics of each patch during the cycle are computed via a Gillespie‐algorithm simulation. This simulation proceeds for a single patch until the end of the cycle; each patch is simulated sequentially, with the order in which each is simulated randomized within each cycle to minimize any effects of priority. We distinguish between 28 classes of events within these small‐scale simulations, each representing the decay, birth, infection, lysis, immigration, or emigration of a category of phage or host, as well as stochastic decay of the two possible resources. The state is defined by nine variables: generalist and specialist phage, uninfected individuals of hosts *A* and *B*, infections between generalists and A, generalists and B, and specialists and A, and the two resources.

Host populations grow with Michaelis–Menten kinetics, with maximum growth rates *r*_*A*_ and *r*_*B*_ and a half‐saturation constant *K* shared by both types. Birth of a single host consumes one unit of resource out of an initial *Y* units. Hosts arrive at a patch with a rate given by the product of a coefficient, *v*, and the abundances of patches and of hosts in the surrounding medium. Hosts do not migrate to a patch without any of their required resource, but otherwise this behavior is independent of the patch's state. Hosts also leave the aggregation according to a sigmoidal rate function, which is $d\left(1-\frac{1}{1+\frac{k}{\left|x\right|}}\right)$ for the resource *x*; these parameters are also shared across both host types. Note that *k* here is distinct from *K*, the half‐saturation constant for host growth. Resources also decay at a rate *σ*; this parameter helps ensure that simulations do not extend indefinitely.

Phage are governed by the parameters previously introduced: a rate of encounter with patches, *ϕ*, a rate of leaving a patch, *α*, an infection coefficient *θ*, and a decay rate *λ*_p_. Infection dynamics are proportional to the product of *θ* and host and phage abundances. Infected cells have exponentially distributed burst times with rate *β*. Host lysis produces a Poisson‐distributed number of progeny phage with means *F*_A_ and *F*_B_ for the two hosts.

We also track four variables to define the surrounding medium—the two phage and the two hosts. Movement of phage and host individuals between this medium and the patches is computed during the simulations of each patch. For computational tractability, we simulate decay of phage and hosts in the medium only once per cycle, after computing the dynamics for each patch. This approximation is coarse‐grained—for example, it subjects a phage that has just arrived in the medium to the same chance of death per cycle as one that has resided there for the entire interval. In a similar spirit, infected cells that migrate out of patches are immediately subject to lysis, releasing their phage progeny into the surrounding medium and avoiding the need to further manage their dynamics.

After processing the decay of individuals in the medium, the algorithm next checks each patch in order to cull any whose resources are near exhaustion. Patches are culled when the remaining quantity of their resources is less than 5% of the starting value, and five or fewer susceptible hosts remain. Once removed from the simulation, any individuals within the patch are moved into the surrounding medium.

Finally, new patches are added according to a schedule, rather than as, for example, a Poisson process; this choice was made to reduce instability of population dynamics, allowing for more consistent results with a more computationally manageable system size. In the *together* treatment, a patch is added after a set number of cycles represented by a parameter *χ*. In the *apart* treatment, two patches are added on the same schedule, one containing only resource *R*_*A*_ and the other containing only *R*_*B*_.

#### 4.2.2 Assessing the suitability of parameter sets

We chose to vary nine parameters affecting phage and host interactions, growth, and death; however, many parameter combinations quickly resulted in extinction of phage or both hosts and, consequently, phage. Additionally, some combinations compatible with coexistence produced dynamics that presented practical issues for our study: strong, undamped oscillation, very slow convergence to equilibria, or small equilibrium abundances that were therefore highly sensitive to demographic stochasticity. Because our goal was to study a parameter set by varying *F*_B_, we needed parameter sets that resulted in well‐behaved dynamics regardless of the value of *F*_B_. We therefore simulated systems of generalist phage, in combination with hosts *A* and *B*, over 20 random values of *F*_B_ drawn from the range 0–25. We then applied heuristic criteria for suitability and stability: after a burn‐in period, we required that the mean abundances of each host and the phage exceed 10,000, that the minimum values across the ensemble of replicates exceed 5000 at each time point, and that the absolute values of slopes of mean abundances be less than 0.5% of the mean values. These values were chosen to balance the need for large, stable populations with the desire to sample the parameters broadly. We did not adjust these criteria to include or exclude any parameter set based on whether the data derived from that parameter set was consistent with our hypotheses.

The burn‐in time, provided to allow initial transient dynamics to settle, was equal to 5 years of simulation time; populations were then measured for an additional 5 years. Populations always began from the same state—1 × 10^5^ of each host and the generalist phage. No doubt some rejected parameter sets would have shown stable coexistence if initiated from a different state, but we judged it impractical to search the space of initial states along with parameter values.

To produce candidate parameter sets, values were drawn from broad, log‐scale distributions. The bounds of these distributions were largely arbitrary, but in some cases were adjusted to attempt to capture the broadest possible range of system behaviors. For example, after we discovered that high values of R*, which were rare in our initial survey, were weakly associated with high values of *ϕ*, we gathered subsequent parameter sets with a higher value of the ceiling of the range of values from which *ϕ* was drawn. Parameter ranges generally varied over three orders of magnitude where biologically reasonable as a way to minimize any effect of the choices of these boundaries.

#### 4.2.3 Inferring R*

To find R*, the ratio of net fecundities at which generalists and specialists have equal fitness, we simulated system dynamics for long periods of time with migration of both generalists and specialists. After an initial period of 5 years, migration introduced small amounts of both phage each cycle. The number of migrants was Poisson‐distributed, with a mean of 2 × 10^−3^ percent of the current number of phage in the surrounding medium. The purpose of low, equal rates of migration was to avoid stochastic extinction of either type, with the aim of capturing a long‐term average frequency best reflecting the outcome of competition between the two strategies. These simulations proceeded for a total of 100 years of simulation time, with data from the last 90 years averaged to produce a mean ratio of the two phage types. A minimum of 25 simulations were performed for each parameter set, though often many more were performed to reduce uncertainty in the inference.

Across experiments with different values of *F*_B_, we plotted the ratio of specialists to total phage. We expected, and generally saw, sigmoidal relationships, with the frequency of specialists tending to be above 0.5 when *F*_B_ was small and eventually declining to values below the midpoint as *F*_B_ increased. We therefore fit a generalized sigmoidal model, $y=a+\frac{f}{1+e^{b\left(F_{B}-c\right)}}$ to these points using the R function nlsLM from the package “minpack.lm.” Here, *a*, *b*, *c* and *f* are the curve‐fitting parameters without correspondence to simulation parameters. Starting parameter values were adjusted to ensure that nonlinear regression worked without serious issue. Inferred midpoints, found by setting the above equation to 0.5 and solving for inferred values, were all checked visually to ensure that any warning messages or fitting issues reported by the function were negligible in their consequences. We also ran the inference procedure repeatedly with different initial values of the *c* parameter. Minor differences in inferred parameters across these different starting points were accounted for by averaging the resulting inferred values of R*; larger differences were used as an indicator that more data were required to achieve a robust inference.

For a minority of parameter sets, we found that mean ratios of specialists were close to 0.5 for all tested values of *F*_B_. We hypothesized that a strong influence of migration might be homogenizing the observed ratios, and repeated the analysis for these parameter sets with one‐fifth the level of migration. This change introduced more variation in the measured ratios, but generally allowed robust inference of R* for these parameter sets.

During the inference process, three kinds of irregularities became apparent that affected a minority of parameter sets. First, host B was sometimes observed to go extinct during a simulation. For most parameter sets, this happened rarely if at all, and we simply excluded those replicates; this exclusion was not observed to have any substantial effect on the inference. For a few parameter sets judged as suitable by the process above, host B went extinct in most of the simulations in which both generalists and specialists were present. We excluded these parameter sets entirely. Only 3 out of 100 parameter sets were excluded for this reason.

Additionally, we found a few parameter sets for which R* was inferred to be quite high—close to one. In these cases, the ratio of specialist to total phage also exhibited a weak signal to noise ratio, perhaps indicating very weak selective differences. These data suggested that host B was not being effectively utilized by the generalist for these parameter sets. To investigate this discrepancy, we performed simulations with only the generalist and the two hosts and recorded how often host B was infected compared to host A. The three parameter sets showing this very high values of R* all showed extremely low utilization of host B, on the order of 1% as often as host A. These three parameter sets were also characterized by high growth rates of host B and low rates of phage entry into patches. We therefore hypothesized that host B was able to rapidly grow, consume its resource, and depart patches before it could typically be infected by the generalist. Although this scenario may be biologically interesting and supports our hypothesis, we found that we could not reliably estimate R* when host‐B utilization was so low, and consequently selective differences between specialists and generalists were so small. We therefore excluded parameter sets for which the ratio of utilization of host B to host A as measured to be less than 0.02. This cutoff was set to retain as many parameter sets as possible for which we could reliably infer R*. Applying this criterion excluded an additional four out of the original 100 parameter sets.

Finally, for a few parameter sets we found the computational burden of inferring R* to be too high in the *apart* treatment—even after performing hundreds of simulations, we could not infer R* with high confidence due to very low signal‐to‐noise. There was no indication that the inclusion of these parameter sets would alter our findings, so after considerable computational effort we excluded an additional nine parameter sets from the *apart* treatment for this reason.

In Figure S2, we show examples of this inference process for 12 parameter sets, varying the simulation interval to assess its effect. In general, we recover very similar results when this interval is varied by small amounts. However, we do note that the interval seems to affect the stability of some parameter sets. This accounts for some areas in Figure S2 in which points are not shown for specific regions and interval lengths. Overall, though, these data suggest that our central results are not highly sensitive to this aspect of the mechanics of our simulation model.

#### 4.2.4 Measuring the opportunity cost

We hypothesized that R* could be predicted by a measure of opportunity cost: here, proportional to the chance that a phage that contacts host B would go on to successfully infect host A if it were to forgo infecting host B. To measure this, we created a version of the simulation code described above. In this version, we tracked only specialist phage, along with “virtual” phage particles. When a specialist phage came into contact with host B, a virtual phage particle was created. We then tracked the fate of these virtual phage particles as a group, measuring how often they successfully found a host‐A individual before decaying. Virtual particles did not actually infect hosts or otherwise compete with “real” phage, thereby serving to probe the system without perturbing it. Measurements took place over a 20‐year period of model time, with a five‐year burn‐in phase and a five‐year buffer period at the end in which no additional virtual particles were produced. This buffer period ensured that the fates of virtual particles were decided before the outcome of the simulation. At least 30 such replicate simulations were performed, and their results averaged, for each parameter set.

## AUTHOR CONTRIBUTIONS

**Jeremy Draghi:** Conceptualization (lead); formal analysis (equal); funding acquisition (lead); investigation (supporting); project administration (lead); software (equal); supervision (lead); validation (equal); visualization (equal); writing – original draft (equal); writing – review and editing (equal). **Evan Zook:** Conceptualization (supporting); formal analysis (equal); investigation (lead); software (equal); validation (equal); visualization (equal); writing – original draft (equal); writing – review and editing (equal).

## CONFLICT OF INTEREST STATEMENT

The authors declare that there are no competing interests.

## Supporting information


Figures S1 and S2.

## Data Availability

All code and simulation data are available at github.com/etldrz/phage‐aggregation and at Dryad at https://doi.org/10.5061/dryad.w9ghx3fzk
