# Supplementary material for: Spatial clustering of hosts can favor specialist parasites
Source: Ecol Evol. 2024 Nov 17;14(11):e70273. doi: 10.1002/ece3.70273 (PMC11570423; doi:10.1002/ece3.70273)
Supplement: Supplementary file 1 — Figures S1 and S2. [file ECE3-14-e70273-s001.pdf]

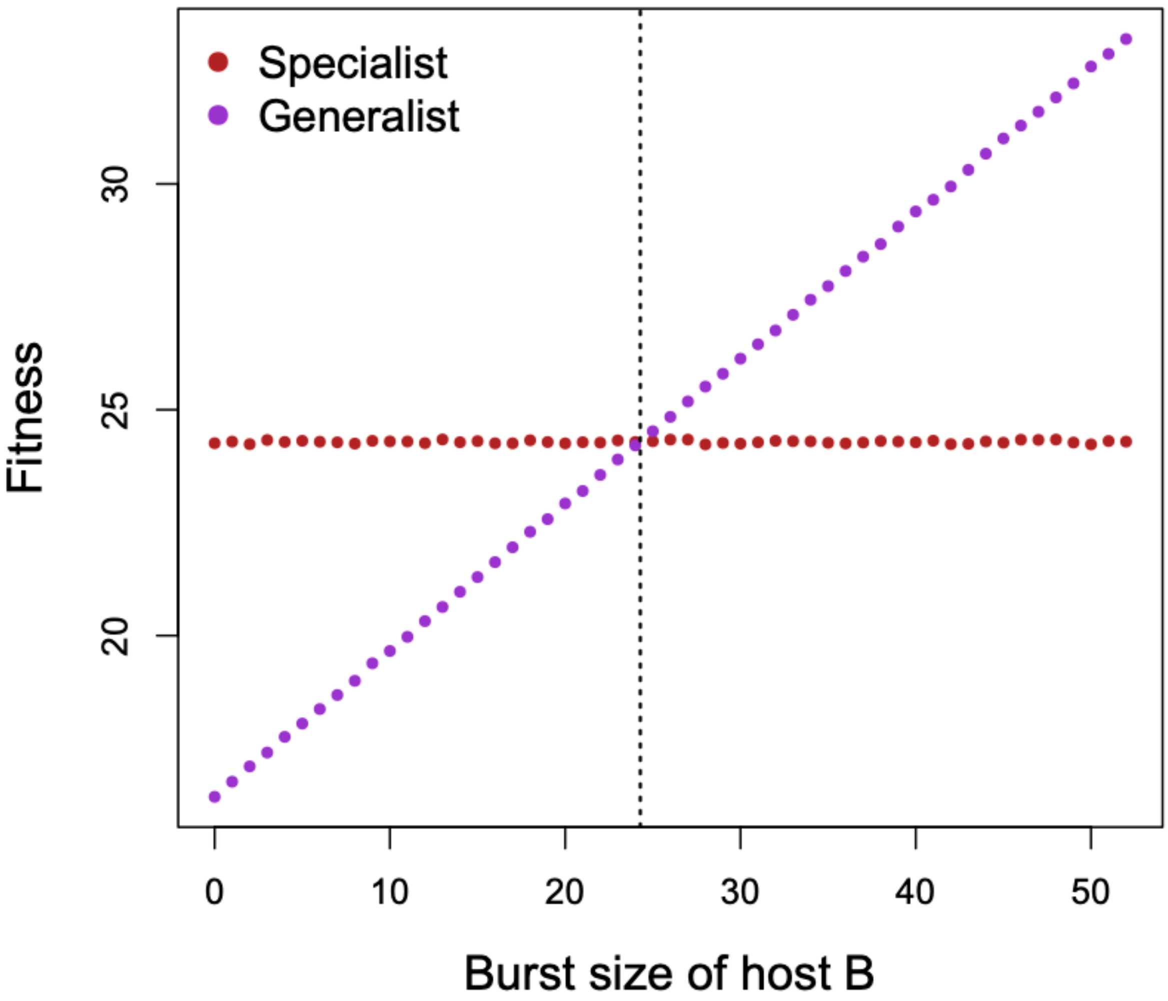

Supplemental Figure 1: Results of simulated phage fitness for the generalist (purple) and specialist (red) types. *R** is defined as ratio of the net burst sizes at the intersection of these lines; the dotted vertical line marks the burst size of host *B* predicted by Eq. 5. The burst size of host *A* is 50, α is 0.1, θ is 0.2, p is 0.5, λ is 0.01, and ω is 0. 10^6 trials were used to simulate each plotted point.

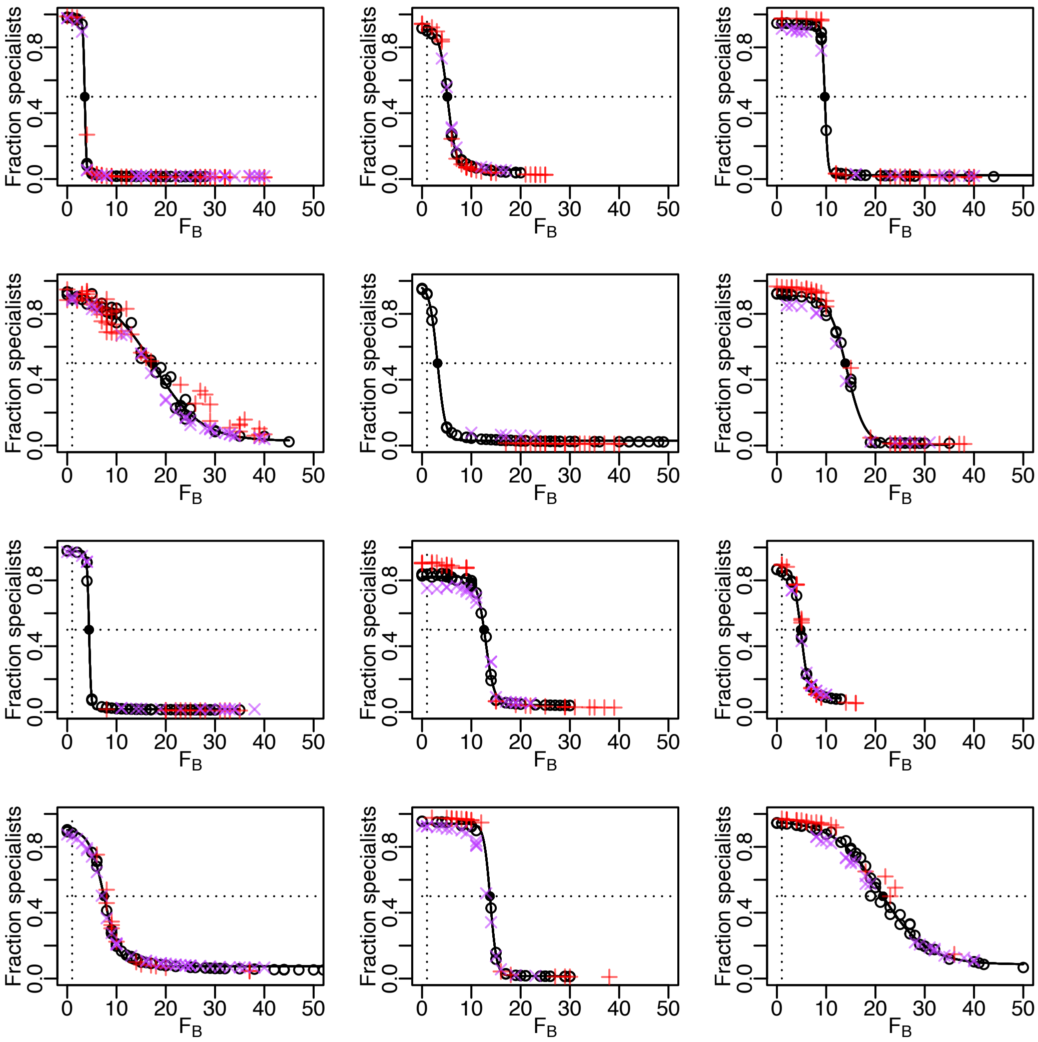

Supplemental Figure 2: Mean frequencies of specialists across ranges of FB for twelve different parameter sets, simulated under the *together* treatment. Black circles depict the default value of a twenty-minute simulation interval, compared to simulations with a thirty-minute (red crosses) or fifteen-minute (purple x's) setting. Parameter sets were chosen to represent a range of values of R*.
